# Supplementary material for: A Liquid Chromatography-Mass Spectrometry Method to Study the Interaction between Membrane Proteins and Low-Molecular-Weight Compound Mixtures
Source: Molecules. 2022 Jul 30;27(15):4889. doi: 10.3390/molecules27154889 (PMC9369908; doi:10.3390/molecules27154889)
Supplement: Supplementary file 1 [file molecules-27-04889-s001.zip › molecules-1840314-Supplementary Figures.pdf]

A Liquid Chromatography-Mass Spectrometry Method to Study the Interaction Between Membrane Proteins and Low-Molecular-Weight Compound Mixtures

Ogiso et al.

Supplementary Material

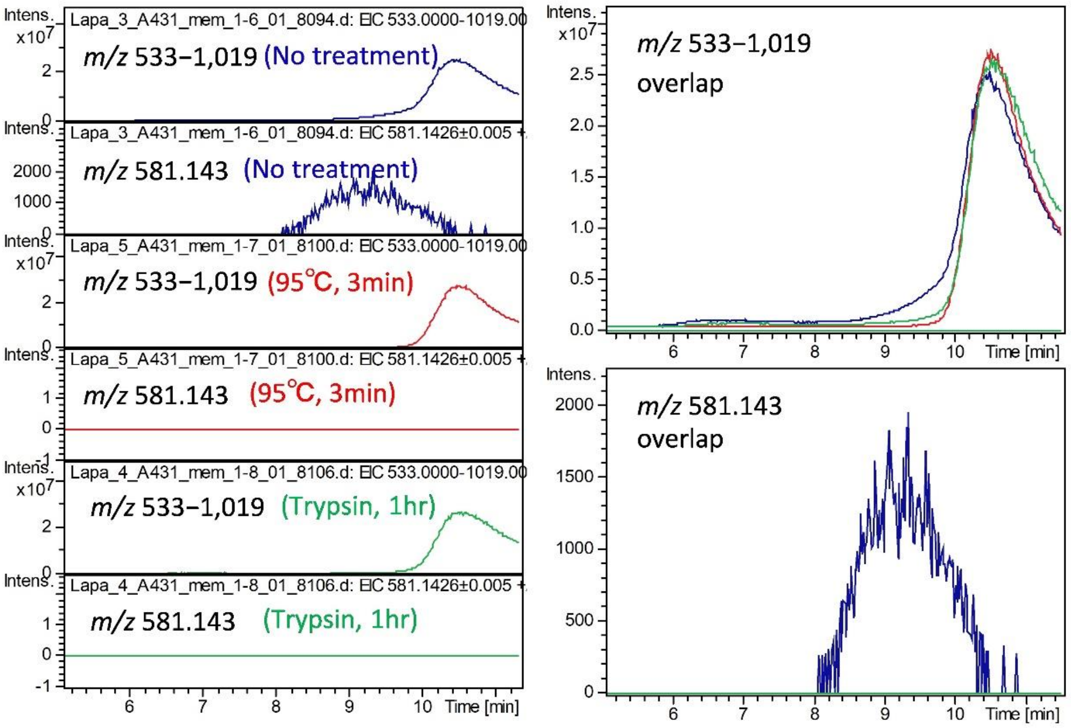

Figure S1. Effects of trypsin and heat treatments of a membrane protein fraction on the lapatinib interaction.

Two μM of lapatinib were added to the membrane protein fraction from A431 or CHO cells, followed by injected into SEC-MS of the mixture. The elution profiles of lapatinib (*m/z* 581.143) were compared when pre-treated membrane protein fractions were used.

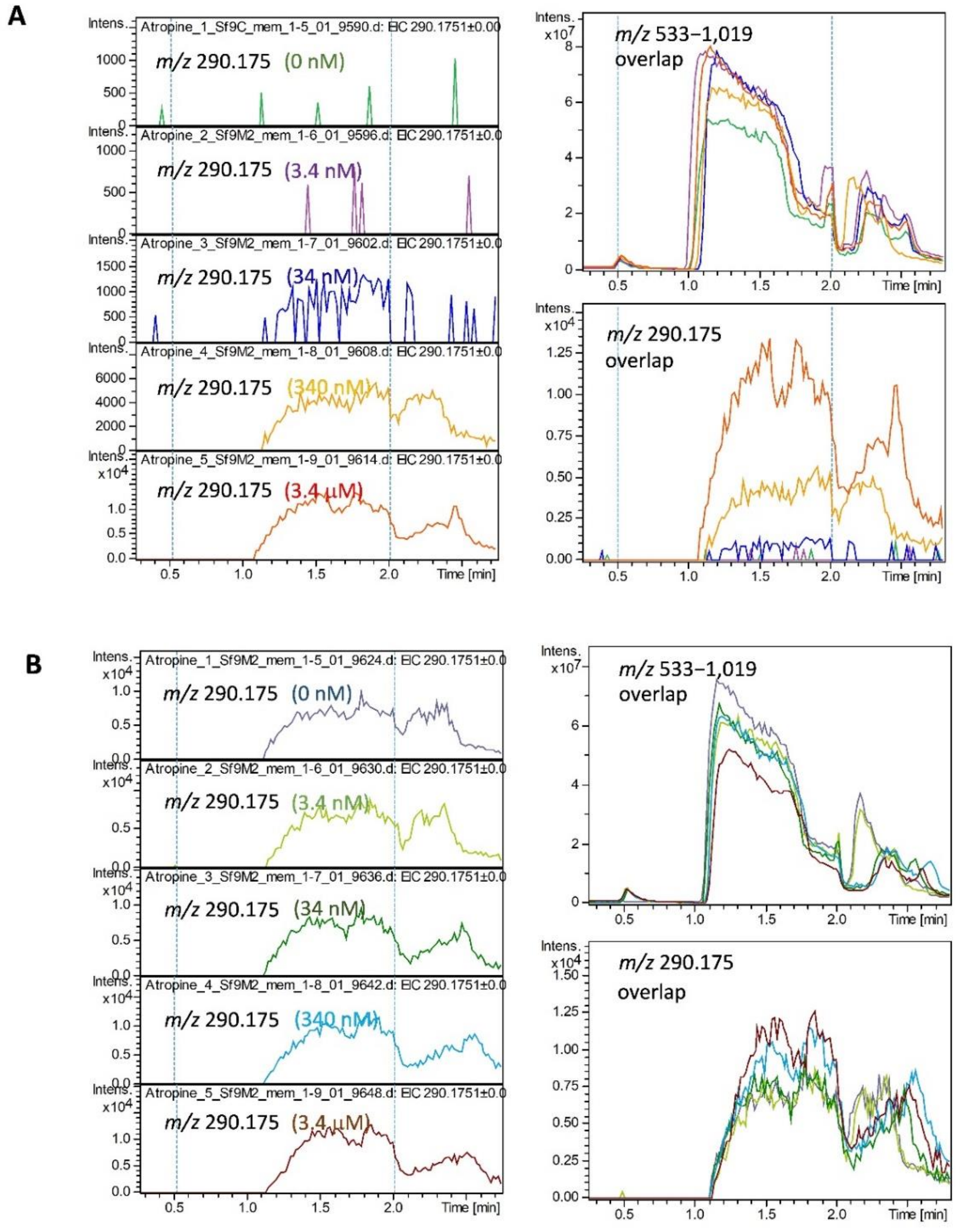

Figure S2. Detection of interaction between the membrane protein fraction from M2R-expressing Sf9 cells and the M2R antagonist, atropine.

(A) Concentration dependence of the atropine interaction. 3.4 nM to 3.4 μM of atropine were added to the membrane protein fraction from M2R-expressing Sf9, then, the interaction between atropine and membrane proteins was analyzed. Membrane proteins were solubilized without the addition of atropine.
(B) Concentration dependence of the atropine interaction. Same as (A) except for that membrane proteins were solubilized with the addition of atropine.

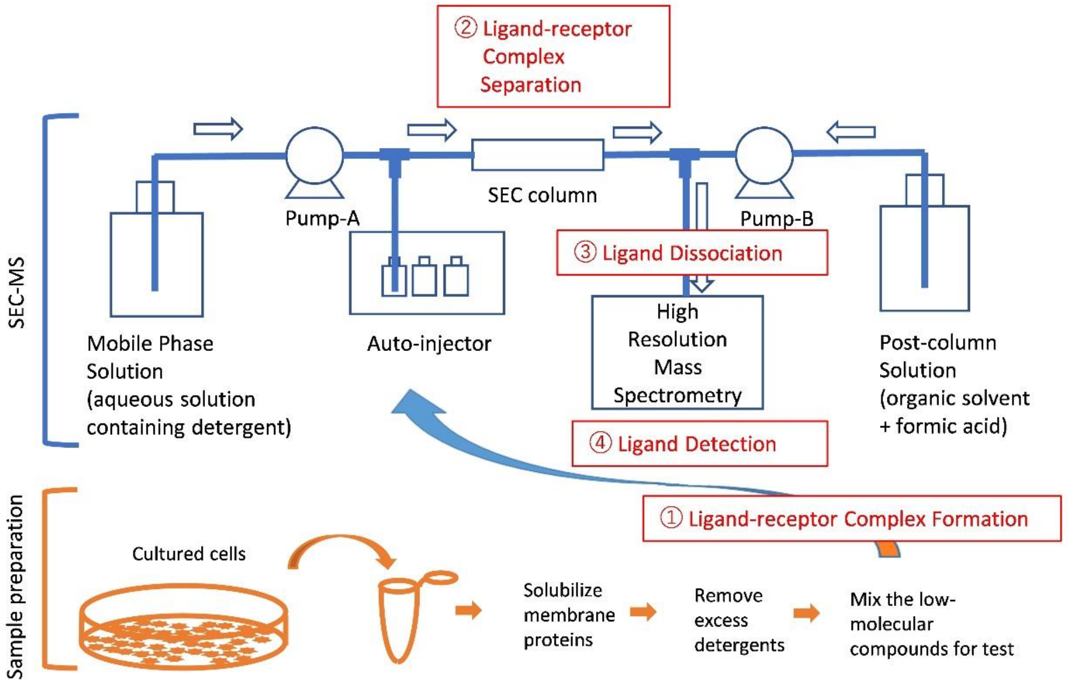

Figure S3. The outline of the SEC-MS method to study the interaction between membrane proteins and LMW compound mixtures.

Membrane protein fractions are obtained from cells cultured on 10 cm-dish, to which a mixture of LMW compounds is added. The formed ligand-receptor complexes are separated from the unbound LMW compounds by SEC, and then the ligands in the complex are dissociated online, and are measured by mass spectrometry.
